# Appealing to Individual Fears or Social Norms: How Can the Public Be Persuaded to Accept COVID-19 Vaccination through Risk Communication?

**DOI:** 10.3390/ijerph192113737

**Published:** 2022-10-22

**Authors:** Fangfei Wang, Sifan Zhang, Lei Lei

**Affiliations:** 1 Faculty of Humanities and Social Sciences, Dalian University of Technology, Dalian 116024, China; 2 School of Journalism and Communication, Minzu University of China, Beijing 100081, China

**Keywords:** COVID-19 vaccination, risk communication, fear appeals, social norms

## Abstract

In the context of the COVID-19 pandemic, improving the public’s understanding of the increased efficacy and safety of the COVID-19 vaccines through scientific risk communication campaigns, promoting the public’s acceptance and willingness to receive COVID-19 vaccines, and forming collective actions at the social level will deeply impact on the effect of COVID-19 prevention in various countries, which is also a key factor that governments need to address urgently. Previous research on risk communication has mostly focused on microscopic perspectives of how to stimulate individual self-protection behaviors by awakening threat and efficacy perceptions; however, a lack of observation of social collective actions means there is a risk of failure regarding COVID-19 epidemic reduction and prevention. In this regard, this study was based on the issue of vaccination in the context of the COVID-19 epidemic through a highly regulated and controlled research experiment in China (*n* = 165), which was designed to examine the impact of two risk communication frameworks, appealing to individual fears and appealing to social norms, on the public’s acceptance and recommendations of COVID-19 vaccines, thus outlining the path of action from individual protection to collective epidemic prevention. Both the “fear appeals” framework and the “social norms” framework were found to have a positive effect on the Chinese public’s vaccination acceptance. Specifically, social norms information may increase vaccination acceptance by enhancing the public’s perceptions of social responsibility, while fear appeals information may reduce their perceptions of threat and social pressure to get the vaccine. Female and highly educated groups were more likely to refuse to recommend vaccination after reading the risk communication information. These results can be a useful supplement to the theory and practice of risk communication.

## 1. Introduction

This is not the first time the World Health Organization (WHO) has responded to a pandemic, nor will it be the last—however, COVID-19 is the most challenging crisis we have ever faced [1], posing major challenges to global public health agencies, as well as greatly disturbing social and economic development around the world. Compared to previous major public health emergencies, this event has a wider scope, faster spread, greater variation, and greater uncertainty for prevention and control. During the prevention and control of the COVID-19 epidemic, in addition to implementing traditional means, such as emergency management, medical security, and the information disclosure, the importance of promoting the COVID-19 vaccination has become increasingly prominent. The World Health Organization (WHO) has clearly stated that COVID-19 vaccines are very effective in substantially reducing severe illness and death from the rapidly mutating virus, with the vaccines containing a high degree of safety and an extremely low risk of serious adverse events [2].

Scholars generally agree that effective risk communication efforts have become the key factor in promoting vaccination. Risk communication research began in the 1980s, intending to design appropriate message frameworks and communication strategies to convey the significance of health or environmental risks, to enable the public to scientifically perceive and assess the risk and take actions to manage and control it [3]. As research progresses, risk communication research has gradually expanded from environmental issues, food safety issues, and natural disasters to health issues such as health risk communication, health beliefs persuasion, and health behaviors promotion. Existing research has, in general, concluded that the public’s actions to deal with risks usually follow the path of “contact information—form perception—take action” [4]. As a result, most of these studies have focused on the micro-individual level, which explores the impact of individual information exposure on an individual’s risk perceptions and self-protection behaviors [5]. Among them, it is recognized by many scholars as an effective means to enhance public risk perception and stimulate self-protection [6]. Emerging in the 1950s, fear appeals are used to induce fear in individuals by presenting threatening negative information, thereby motivating them to accept the persuasive information transmitted; the theory has been enriched and extended during this time. A more recent and representative model is the “Extended Parallel Process Model” (EPPM) proposed by Witte, which indicates that the public’s assessment of fear appeals ranges from “perceived threat” (such as severity and susceptibility) to “perceived efficacy” (such as self-efficacy and response efficacy). Furthermore, three modes of action were identified: “no response”, the “danger control process” (making adaptive changes), and the “fear control process” (such as refusing to accept fear appeals) [3]. In recent years, there has been an increase in research in risk communication at the micro level, and findings have been applied and verified during the dissemination of various health beliefs and behaviors, such as AIDS prevention and control [7], breast self-examination [8], skin cancer prevention [9], hearing protection [10], quitting e-cigarettes [11], as well as vaccinations [12,13].

Overall, previous research suggests that appealing to an individual’s fear is related to their health behaviors, such as vaccinations [12,14]. In contrast, risk communication research has paid less attention to the macro-level, which promotes the integral participation of social forces and collective action in risk situations, leading to the mechanism linking risk communication to collective actions being overlooked. However, in the context of preventing and controlling COVID-19, where collective actions (such as self-protection and vaccination) need to be fostered among the majority of the public, it is difficult to provide operational guidelines for risk communication practices. Although the WHO has actively promoted the “COVID-19 Vaccine Implementation Plan”, 28 nationally representative samples from 13 countries showed that as the COVID-19 pandemic progressed, the proportion of people who intended to be vaccinated decreased, while the proportion of those who planned to refuse vaccination increased [15]. As of 13 January 2022, 36 WHO member states had a COVID-19 vaccination rate of less than 10% and 88 member states had a coverage rate of less than 40% [16]. In China, however, a very different picture emerges. Compared to most countries, in China, the epidemic is generally controllable and the threat is relatively low, but the public has a higher degree of recognition and willingness to get the COVID-19 vaccine. A survey of the Chinese public showed that 97% of the respondents had received at least one shot of the COVID-19 vaccine [12]. The reasons for this are closely linked to the effective risk communication practices conducted by Chinese governments in promoting the COVID-19 vaccination. Specifically, based on the characteristics of China’s socio-cultural environment, the Chinese government has introduced a “social norms” framework in their communication practice, which describes vaccination as a social responsibility and that the majority of the public has been vaccinated. This introduces new perspectives on risk communication research.

In response, this study conducted a controlled experiment in China from November 2021 to January 2022, to examine the impact of two risk communication frameworks, appealing to individual fears and appealing to collective social norms, on the public’s acceptance and recommendations of the COVID-19 vaccination. On this basis, the study outlined the pathways through which risk communication frameworks influenced the public’s scientific perception of vaccine risks, acceptance of vaccines, and recommendation of vaccination to others, thus exploring the mechanisms of action from individual protection to collective epidemic prevention. Thus, the findings can provide insights into the promotion of COVID-19 vaccines and risk communication studies in the future.

## 2. Materials and Methods

### 2.1. Experiment Design

**Figure 1:**
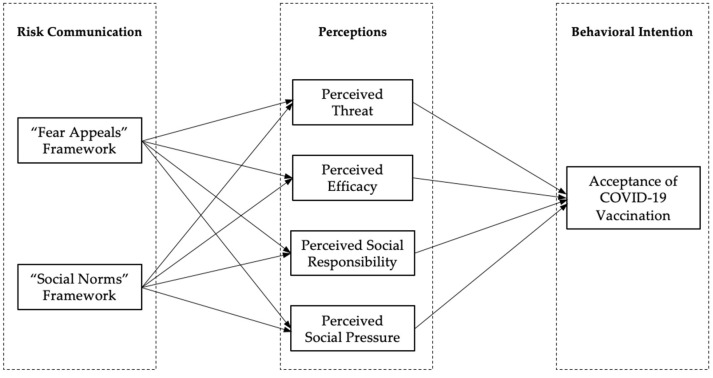
Hypothetical model.

Combining the findings of existing risk communication studies and China’s communication experience with COVID-19 vaccines, this study aims to examine the influence of two risk communication frameworks, appealing to individual fears and appealing to the collective social norms, on the public’s acceptance of the COVID-19 vaccination. According to the EPPM model of fear appeals theory, the “fear appeals” framework includes two core elements of “threat” and “efficacy” [17]. Referring to social norms theory and China’s risk communication practices, the “social norms” framework includes two items of “social responsibility” and “social pressure” [18,19]. The theoretical model diagram is shown in Figure 1.

Specifically, this study designed a controlled experiment to directly observe the impact of two risk communication frameworks. In designing the content of risk communication, both frameworks referred to Chinese media reports to ensure that the content was scientific and reliable. The fear appeals framework aimed to highlight the threat of the COVID-19 epidemic and the effectiveness of vaccination. Among them, the threat information referred to the susceptibility and severity of SARS-CoV-2, and the damage to people’s bodies and property; conversely, the efficacy information emphasized the effectiveness of vaccines against SARS-CoV-2, which is also a core issue of media reports on the COVID-19 epidemic. For example, the experimental materials contained statements such as “the cumulative number of confirmed cases of COVID-19 epidemic worldwide reached 249,743,428, an increase of 317,865 from the previous day, and 5,047,652 deaths, safe and effective vaccines are the most powerful weapon against the SARS-CoV-2, which can stimulate the human body to develop immunity against the COVID-19 epidemic and block SARS-CoV-2 infection”. The social norms information emphasized that vaccination was not just an individual protection behavior, but a national responsibility and a social obligation. For example, the experimental materials indicated that “Every citizen has to cooperate with the national epidemic prevention work and take the initiative to vaccinate. Many compatriots have already done their part to build a group defense line”.

In addition, to ensure the effectiveness and accuracy of the experiment, the experiment was designed with the following guidelines: (a) The number of words in the text of the two experimental materials was controlled within the range of 400–500 words, to ensure the same amount of information in each experimental material. (b) In addition to the two experimental groups, a reference group was set up. Subjects in the reference group did not read any information about COVID-19 vaccines, but only answered questions about their cognition and attitude toward vaccination. Thus, the values of the dependent variable in the reference group can reflect the public’s general attitudes and behavioral intentions towards COVID-19 vaccines, while changes in the values of the dependent variable in the two experimental groups can assess the immediate effects caused by different risk communication frameworks. The specific experimental materials used in the study are shown in Appendix A.

### 2.2. Measures

#### 2.2.1. Perceived Threat and Perceived Efficacy

Perceived threat and perceived efficacy involve the public’s appraisal of the characteristics of the “fear appeals” framework and form the starting point for their subsequent attitude and behavioral intention. To assess the perceived threat and perceived efficacy, we adopted Witte’s Risk Behavior Diagnosis Scale (RBD) [17], while integrating the context of the COVID-19 epidemic by making suitable adjustments. The measurement of perceived threat was directly assessed using a statement: “Everyone is at risk of being infected by SARS-CoV-2.” The assessment of perceived efficacy was described as “the third booster shot of COVID-19 vaccines is the most effective way to prevent the epidemic”. All the responses were based on a 5-point Likert scale (1 = strongly disagree, 5 = strongly agree).

#### 2.2.2. Perceived Social Responsibility and Perceived Social Pressure

Perceived social responsibility and perceived social pressure involve the public’s appraisal of the characteristics of the “social norms” framework and form the starting point for their subsequent attitude and behavioral intention. The assessment of these two variables was based on the research of Cialdini and Lapinski [18,19], and was adapted according to the actual situation. Perceived social responsibility was described as “it is the duty and obligation of citizens to get the COVID-19 vaccine, especially a third booster shot of the COVID-19 vaccine”. Perceived social pressure involved an evaluation of peer pressure—“most people have received the primary shots of COVID-19 vaccines and will continue to receive the third shot”—to persuade the public to conform and do the same thing. Responses were based on a 5-point Likert scale (1 = strongly disagree, 5 = strongly agree).

#### 2.2.3. Acceptance of the COVID-19 Vaccination

The following considerations were made regarding the social reality in which this study was conducted: (a) Because of the strong publicity campaign and vaccination policy adopted by the Chinese government to promote COVID-19 vaccines, the rate of the Chinese public’s vaccination on primary shots had already reached a high level. (b) The Chinese government was in the process of promoting the third shot of the COVID-19 vaccine, and because the epidemic was largely under control in China, parts of the public doubted the value of the third shot and were less willing to get vaccinated [20]. (c) Chinese practical experience in preventing and controlling the COVID-19 epidemic showed that the key to success lies not only in the promotion of individual health behaviors, but also in the formation of collective action.

Therefore, this study focused on the recommendation of a third shot of the COVID-19 vaccines to evaluate the public’s acceptance of the COVID-19 vaccination. On this basis, the results of this study can be inferred from the pathways through which risk communication had an influence on awakening individual willingness to receive the COVID-19 vaccine and forming the social collective vaccination.

As for measurements, the variable was described as “I will persuade others to receive a third shot of COVID-19 vaccine”, and assessed using a scale of “disagree”, “somewhat agree”, and “agree”.

#### 2.2.4. Control Variables

Referring to previous studies [21], this study used the following factors as control variables: gender (1 = male, 2 = female), age (from “16–20” to “over 60”, in ascending order of 1–6), educational experience (from “high school graduate” to “postgraduate”, in ascending order of 1–4), monthly income (from “no income” to “more than CNY 20,000”, in ascending order of 1–6), and regional risk level (evaluated with whether the area you live in has been classified as a medium-high risk area). All the survey variables used in the regressions are included in Appendix B.

### 2.3. Experiment Procedure

The experiment was carried out as a network control experiment. The questionnaire was distributed on a Chinese survey platform, Credamo, and the participants were recruited by professional research companies. The recruitment period was from November 2021 to January 2022.

Based on the high rate of the COVID-19 vaccination in China, this study selected the public who have been vaccinated with the primary shots of COVID-19 vaccines as the subjects. Because they have recognized the value of the COVID-19 vaccines to some extent, their changes in attitudes and behavioral intentions that occurred after reading the experimental materials can directly reflect the persuasive effects of risk communication frameworks.

After completing the online consent form, the subjects were asked about their vaccination status: “Have you gotten the primary shots of the COVID-19 vaccine?” Only subjects who answered “yes” were allowed to participate in this experiment. In the experiment, subjects were randomly assigned by the system to one of three experimental situations (“fear appeals” group, “social norms” group, or reference group), and their reading time was recorded in the background of the questionnaire to control the validity of the sample. Afterward, the subjects were asked about threat perception, effectiveness perception, perceived social responsibility, perceived social pressure, and their general acceptance of the COVID-19 vaccination. A total of 165 valid samples, with 55 samples in each group, were obtained for the experiment. The demographic indicators are shown in Appendix C.

### 2.4. Data Analysis

Power analysis was conducted to assess the statistical power of the experiment, using the “pwr” package in the R software. Results showed that the original sample of 165 (with 3 groups, 55 in each group) had an α err. Prob. = 0.05 and a power (1−β err. Prob.) = 0.9; the effect size was of an approximately medium range, with a Cohen’s f = 0.22 [22].

Other statistical analyses were conducted using the Stata Special Edition 15.1 statistical software, created by StataCorp in College Station, TX, USA. The data were described using frequency counts and percentages. We conducted linear regression analyses to assess the impact of risk communication frameworks and other factors on the acceptance of the COVID-19 vaccination. In the first step, we introduced only risk communication frameworks and control variables to predict the public’s vaccination willingness and intentions to recommend vaccination. Then, we conducted path analysis to outline the pathways through which risk communication frameworks can make an influence. The perceived threat, perceived efficacy, perceived social responsibility, and perceived social pressure were in turn analyzed as dependent variables in linear regression models.

### 2.5. Ethics Statement

Although ethical review and approval were not required for the study on human participants by the local legislation, this study followed strict academic ethical guidelines: the experiment was conducted under the guidance of the ethics committee in the authors’ institution. Participation in the experiment was voluntary. Each respondent was provided with sufficient information about the experiment to make an informed choice on whether or not to participate. The data collected were anonymous and confidential.

## 3. Results

### 3.1. Impact of “Fear Appeals” and “Social Norms” Frameworks on the COVID-19 Vaccination Recommendation

As noted, attitudes and behavioral intentions towards vaccination might be directly influenced by different risk communication frameworks and demographic characteristics. In a linear multiple regression model, the fear appeals framework, the social norms framework, and control variables were regressed on the recommendation of the COVID-19 vaccination (Table 1). After controlling for other variables in the model, both the “fear appeals” framework and the “social norms” framework were found to be statistically significant (*p* < 0.1). Compared to samples in the reference group who did not read any information, samples who read information about “fear appeals” (B = 0.191, *p* = 0.043) and “social norms” (B = 0.169, *p* = 0.082) were more likely to recommend others to get the COVID-19 vaccine. Among the control variables, female gender (B = −0.213, *p* = 0.006) and having an advanced degree (B = −0.143, *p* = 0.031) had a significant negative relationship with the public’s vaccination recommendation. 

### 3.2. Mechanism Linked between Risk Communication Frameworks and Acceptance of the COVID-19 Vaccination

**Figure 2:**
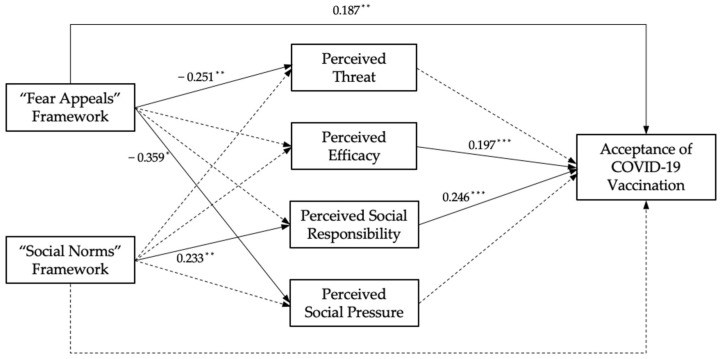
Hypothetical model test results, where solid lines represent established paths, and dashed lines represent untenable paths. All the established paths are marked with the effect value (B) (* *p* < 0.1, ** *p* < 0.05, *** *p* < 0.001).

In the pathway analysis, perceived threat, perceived efficacy, perceived social responsibility, perceived social pressure, and acceptance of vaccination were in turn analyzed as dependent variables. The results are shown in Figure 2. The hypothetical model is partially established.

In the first step, linear regression analysis was conducted to test the relationship between risk communication frameworks and the public’s perceptions. After controlling for other variables, results showed that the perceptions of the samples who read risk communication information were significantly different from those in the reference group. Specifically, after reading fear appeals information, there was a significant decrease in samples’ perceptions of threat (B = −0.251, *p* = 0.024) and social pressure to get vaccinated (B = −0.359, *p* = 0.030), with no change in the perceived efficacy (B = 0.011, *p* = 0.944) or perceived social responsibility (B = −0.042, *p* = 0.705). Conversely, after reading social norms information, there was a significant increase in samples’ perceptions of social responsibility to get vaccinated (B = 0.233, *p* = 0.0.082), with no change in the perceived threat (B = −0.143, *p* = 0.208), perceived efficacy (B = −0.099, *p* = 0.550), or perceived social pressure (B = −0.053, *p* = 0.752).

In the second step, both risk communication frameworks and the public’s perceptions, along with the control variables, were regressed on the recommendation of the COVID-19 vaccination. Among all the independent variables, fear appeals framework (B = 0.187, *p* = 0.030), perceived efficacy (B = 0.197, *p* = 0.000), and perceived social responsibility (B = 0.246, *p* = 0.000) were found to be statistically significant.

Then, we conducted a Goodman test to estimate the mediation effects. Results showed that perceived social responsibility was a significant mediator in the relationship between the social norms framework and the acceptance of vaccination (*p* < 0.1).

## 4. Discussion

Referring to previous risk communication studies and the Chinese government’s successful practices during the COVID-19 epidemic, this study aims to determine the relationship between risk communication information and collective actions to prevent and control the epidemic. Specifically, a controlled experiment was conducted in China from November 2021 to January 2022, taking the promotion of the third dose of the COVID-19 vaccines as the context. In the experiment, the “social norms” framework was introduced along with the “fear appeals” framework, which had been widely used in promoting vaccination uptake, and the COVID-19 vaccination recommendation was used as the indicator to evaluate collective vaccination. Furthermore, the public’s perceptions were divided into four dimensions—perceived threat, perceived efficacy, perceived social responsibility, and perceived social pressure—and the mediating effect of the four perceptions was investigated.

First, both the “fear appeals” framework and the “social norms” framework were found to have a positive effect on the Chinese public’s acceptance of the COVID-19 vaccination. The success of the “fear appeals” framework in promoting vaccines was consistent with the findings of previous studies [23,24,25]. Conversely, the significant effect of the “social norms” framework provided new empirical evidence for risk communication studies. It also confirmed previous findings on the reasons for the success in preventing and controlling the COVID-19 epidemic in East Asian countries [26,27,28]. In East Asian countries, the public is deeply influenced by Confucianism, thus making them have a stronger collective consciousness and more willingness to follow social norms. During the COVID-19 epidemic, the public is more willing to cooperate with the government’s policies and criticize violators [29]. These results show that it is necessary to adjust strategies of risk communication by closely integrating the socio-cultural background and the values of the audience.

Second, this study refined the mechanisms of different risk communication frameworks on the public’s attitudes and behavioral intentions towards the COVID-19 vaccination program and obtained interesting findings. It was found that different risk communication frameworks could construct very different perceptions of the COVID-19 epidemic and vaccines. Participants who read the social norms information had a significantly higher perception of social responsibility to get vaccinated, which is consistent with the theoretical assumptions and research design of this study. However, for participants who read fear appeals information, there was a significant decrease in the perceived threat of epidemic and perceived social pressure to get vaccinated, and no change in the perceptions of efficacy and social responsibility. Moreover, participants’ perceived efficacy and perceived social responsibility significantly promoted their recommendation of the COVID-19 vaccines, while perceived threat and perceived social pressure were not significant. These results may be related to the psychological status and risk knowledge of the public. With the continued spread of the epidemic, most of the public is under greater stress [29], leading to an increased motivation to avoid negative information [30]. In addition, there is a significant increase in the public’s knowledge of SARS-CoV-2. As a result, they prefer positive guidelines on health behavior rather than negative information. When exposed to large amounts of fear appeals information, they are more inclined to reject it or take antagonistic interpretations, thus making information related to the threat or social pressure ineffective. Previous studies also found that the gain framework was more effective under low uncertainty conditions, while the loss framework was more persuasive under high uncertainty conditions [31]. The implication is that risk communication information on the COVID-19 vaccines should be refined to the public’s psychological status and existing risk knowledge.

Third, this study looked at whether the demographic characteristics were related to the acceptance of COVID-19 vaccines. The results showed that female and highly educated participants were less likely to recommend vaccines after reading risk communication information. This may be related to their higher safety concerns on potential negative health outcomes of COVID-19 vaccines, which has been supported by other published studies [32]. Therefore, when promoting the COVID-19 vaccination, it is necessary to provide detailed information about the vaccines’ side effects in addition to emphasizing the effectiveness, especially to people who are sensitive to vaccine safety issues.

### Limitations

However, there are a few limitations in this study. First, although the controlled experiment based on the online questionnaire is convenient and able to establish cause-and- effect relationships between risk communication information and vaccination acceptance, there are inherent limitations of the study design, such as the small sample size and a low proportion of elderly participants due to their poor skills in accessing the online survey. Second, considering that vaccination acceptance is a complex and multifactorial construct [33], other factors associated with the public’s attitudes towards the COVID-19 vaccines need to be introduced, such as risk knowledge. Third, the study was conducted in China, and Chinese-specific socio-cultural context limits the applicability of the findings to other countries. In the future, additional studies in more countries, with larger samples and more assessment factors are needed, in order to further understand the interplay of risk communication information and cognitive and demographic factors associated with COVID-19 vaccination acceptance.

## 5. Conclusions

This study examined the cause-and-effect relationships between risk communication information and the public’s perceptions and acceptance of the COVID-19 vaccination, based on a controlled experiment. The findings suggested that Chinese people could be effectively persuaded to accept the COVID-19 vaccines by information appealing to fears or social norms. Specifically, social norms information may motivate the public to recommend the COVID-19 vaccines by enhancing their perceptions of social responsibility, while fear appeals information may reduce their perceptions of threat and social pressure to get the vaccine. Among the demographic characteristics, gender and education experience were found to have a significantly negative effect, implying that female and highly educated groups were more likely to refuse to recommend vaccination after reading the risk communication information. Therefore, it is necessary to focus more on the public’s cognitive and demographic characteristics, such as psychological status, risk knowledge, and safety concerns, when conducting risk communication on the COVID-19 vaccines. Only then will governments be able to design segmented risk communication contents and optimal communication programs which will effectively increase vaccination acceptance.

## Figures and Tables

**Table 1:** Linear regression model of vaccination recommendation on risk communication frameworks, *n* = 165.

Variable	B	*p*-Value	[95% CI]	
“Fear Appeals” framework	0.191	0.043	0.006	0.377
“Social Norms” framework	0.169	0.082	−0.022	0.359
gender	−0.213	0.006	−0.362	−0.063
age	0.02	0.757	−0.108	0.149
education experience	−0.143	0.031	−0.273	−0.13
monthly income	0.054	0.187	−0.027	0.135
regional risk level	0.002	0.984	−0.169	0.173

*R*^2^ = 0.1180.

## Data Availability

The data presented in this study are available on request from the corresponding authors. The data are not publicly available due to participants’ privacy concerns.

## Appendix A

**Table A1:** Details of Experimental Materials.

Experimental Group	Reference Document	Details
Fear appeal	Refer to articles on Euronews online and the National Health Commission of the People’s Republic of China website	This round of the epidemic has affected 20 provinces, involving multiple ports and transmission chains.
		According to the official website of the World Health Organization, the cumulative number of confirmed cases of SARS-CoV-2 worldwide reached 249,743,428, an increase of 317,865 from the previous day, and 5,047,652 deaths. The World Health Organization has warned that 500,000 people could die from the outbreak this winter amid a surge in confirmed cases of SARS-CoV-2 in Europe and slow progress in vaccination efforts in some regions [34]. Liangyou Wu, deputy director of the National Health Commission’s Bureau of Disease Control and Prevention, pointed out that from 17 October to 5 November at 24:00, there have been 918 cases of infection across the country, and the current round of the epidemic has spread to 20 provinces. With the characteristics of rapidity and wide range, the prevention and control situation was severe and complicated [35]. A safe and effective vaccine is the most powerful weapon to prevent the virus, which can stimulate the human body to produce immunity against SARS-CoV-2 and block the virus infection. At present, the overall protection rate of the COVID-19 vaccine against the mutant strain is 59%. Since the protection rate of the vaccine is not 100%, it takes about 80–85% of the public to be vaccinated to establish the herd immunity barrier, so please get the COVID-19 vaccine as soon as possible!
Social norms	Refer to articles on the National Health Commission of the People’s Republic of China website	Vaccination against SARS-CoV-2 is a national responsibility and a social obligation! Please cherish the opportunity to be vaccinated!
		Dear friends: It is the obligatory responsibility of every citizen to cooperate with the national epidemic prevention work and to actively get vaccinated. Because you have a strong motherland, you can enjoy the treatment of free COVID-19 vaccine! Everyone should be grateful to grow up in this great country, and be grateful to the people who selflessly sacrificed for vaccination. The whole country is fighting against the epidemic, and what each of us can do is to actively respond to the call and get vaccinated against the COVID-19 epidemic as soon as possible. Many people have already done their best to build a group defense line. China’s COVID-19 vaccination has reached 2.3 billion doses, and more than 1 billion people have been vaccinated [36]. However, since the protection rate of vaccines is not 100%, the herd immunity can only be assumed after an 80–85% vaccination rate. Vaccination is a national responsibility and a social obligation! Please get vaccinated as soon as possible to avoid weakening the immune barrier due to your own concerns.

## Appendix B

**Table A2:** Survey Variables Used in Regression Models.

**Age:** What is your age?	
16–20	1
21–30	2
31–40	3
41–50	4
51–60	5
Over 60	6
**Gender:** What is your gender?	
Male	1
Female	2
**Education experience:** What is the highest grade or year of school you completed?	
High School Graduate	1
Technical School	2
College Graduate	3
Postgraduate	4
**Monthly income:** What is your average monthly income (Chinese yuan)?	
No income	1
Lower than CNY 2000	2
CNY 2001–5000	3
CNY 5001–10,000	4
CNY 10,001–20,000	5
More than CNY 20,000	6
**Regional risk level:** After the emergence of Delta virus (August 2021–present), has your area been classified as a medium-high risk area?	
Yes	1
No	2
**Perceived Threat:** Everyone is at risk of being infected by SARS-CoV-2.	
Strongly agree, Agree, No opinion, Disagree, Strongly disagree	1, 2, 3, 4, 5
**Perceived Efficacy:** The third booster shot of COVID-19 vaccines is the most effective way to prevent the epidemic.	
Strongly agree, Agree, Have no opinion, Disagree, Strongly disagree	1, 2, 3, 4, 5
**Perceived Social Responsibility:** It is the duty and obligation of citizens to get the COVID-19 vaccine, especially a third booster shot of the COVID-19 vaccine.	
Strongly agree, Agree, Have no opinion, Disagree, Strongly disagree	1, 2, 3, 4, 5
**Perceived Social Pressure:** Most people have received the primary shots of COVID-19 vaccines and will continue to receive the third shot.	
Strongly agree, Agree, Have no opinion, Disagree, Strongly disagree	1, 2, 3, 4, 5
**Acceptance of the COVID-19 Vaccination:** I will persuade others to receive a third shot of the COVID-19 vaccine.	
Disagree, Somewhat agree, Agree	0, 1, 2

## Appendix C

**Table A3:** Demographic Characteristics of the Sample, *n* = 165.

Variable	Category	*n* (%)
Gender	Female	82 (49.7)
	Male	83 (50.3)
Age	16–20	24 (14.5)
	21–30	78 (47.2)
	31–40	54 (32.7)
	41–50	7 (4.3)
	51–60	2 (1.3)
	over 60	0 (0)
Education experience	High School Graduate	10 (6.0)
	Technical School	27 (16.4)
	College Graduate	115 (69.7)
	Postgraduate	13 (7.9)
Monthly income	No income	13 (7.9)
	Lower than CNY 2000	27 (16.3)
	CNY 2001–5000	20 (12.1)
	CNY 5001–10,000	76 (46.1)
	CNY 10,001–20,000	25 (15.2)
	More than CNY 20,000	4 (2.4)

## Appendix D. Pathway Analysis Results

**Table A4:** Linear regression model of perceived threat on risk communication frameworks, *n* = 165.

Variable	B	*p*-Value	[95% CI]	
“Fear Appeals” framework	-0.142	0.208	−0.469	−0.333
“Social Norms” framework	−0.251	0.024	−0.365	0.08
gender	0.064	0.468	−0.11	0.239
age	0.074	0.333	−0.076	0.224
education experience	−0.027	0.72	−0.18	0.124
monthly income	0.05	0.292	−0.044	0.145
regional risk level	0.099	0.329	−0.101	0.3

*R*^2^ = 0.0931.

**Table A5:** Linear regression model of perceived efficacy on risk communication frameworks, *n* = 165.

Variable	B	*p*-Value	[95% CI]	
“Fear Appeals” framework	0.011	0.944	−0.306	0.329
“Social Norms” framework	−0.098	0.55	−0.424	0.226
gender	−0.103	0.425	−0.359	0.152
age	0.056	0.612	−0.163	0.276
education experience	−0.027	0.806	−0.25	0.194
monthly income	0.096	0.172	−0.042	0.234
regional risk level	0.013	0.925	−0.279	0.307

*R*^2^ = 0.0466.

**Table A6:** Linear regression model of perceived social responsibility on risk communication frameworks, *n* = 165.

Variable	B	*p*-Value	[95% CI]	
“Fear Appeals” framework	0.001	0.991	−0.235	0.238
“Social Norms” framework	−0.011	0.927	−0.253	0.231
gender	−0.192	0.048	−0.382	−0.001
age	0.091	0.272	−0.072	0.255
education experience	−0.076	0.36	−0.242	0.088
monthly income	0.017	0.741	−0.085	0.12
regional risk level	0.227	0.042	0.008	0.445

*R*^2^ = 0.0770.

**Table A7:** Linear regression model of perceived social pressure on risk communication frameworks, *n* = 165.

Variable	B	*p*-Value	[95% CI]	
“Fear Appeals” framework	−0.359	0.03	−0.683	−0.035
“Social Norms” framework	−0.053	0.752	−0.384	0.278
gender	−0.099	0.451	−0.36	0.16
age	−0.083	0.461	−0.307	0.14
education experience	−0.111	0.333	−0.337	0.115
monthly income	0.102	0.151	−0.038	0.243
regional risk level	−0.075	0.621	−0.374	0.223

*R*^2^ = 0.0532.

**Table A8:** Linear regression model of vaccination recommendation on risk communication frameworks and mediators, *n* = 165.

Variable	B	*p*-Value	[95% CI]	
“Fear Appeals” framework	0.186	0.03	0.018	0.354
“Social Norms” framework	0.12	0.162	−0.048	0.29
perceived threat	−0.077	0.207	−0.198	0.043
perceived efficacy	0.196	0	0.112	0.28
perceived social responsibility	0.245	0	0.135	0.356
perceived social pressure	0.018	0.682	−0.069	0.105
gender	−0.193	0.004	−0.325	−0.062
age	−0.024	0.676	−0.138	0.09
education experience	−0.114	0.048	−0.228	−0.001
monthly income	0.045	0.211	−0.026	0.117
regional risk level	−0.043	0.575	−0.195	0.108

*R*^2^ = 0.3454.

## References

[1] WHO WHO Director-General’s Opening Remarks at the Media Briefing on COVID-19—5 February 2021. https://www.who.int/director-general/speeches/detail/who-director-general-s-opening-remarks-at-the-media-briefing-on-covid-19-5-february-2021.

[2] WHO Coronavirus Disease (COVID-19): Vaccines Safety. https://www.who.int/news-room/q-a-detail/coronavirus-disease-(covid-19)-vaccines-safety.

[3] Witte K., Meyer G., Martell D. (2001). Effective Health Risk Messages: A Step-by-Step Guide.

[4] Karlsson P. (2008). Explaining Small Effects of Information-Based Drug Prevention: The Importance of Considering Preintervention Levels in Risk Perceptions. J. Alcohol Drug Educ..

[5] So J., Kuang K., Cho H. (2019). Information Seeking Upon Exposure to Risk Messages: Predictors, Outcomes, and Mediating Roles of Health Information Seeking. Commun. Res..

[6] Dillard J.P., Nabi R.L. (2006). The persuasive influence of emotion in cancer prevention and detection messages. J. Commun..

[7] Bekalu M.A., Eggermont S., Viswanath K. (2017). HIV/AIDS Communication Inequalities and Associated Cognitive and Affective Outcomes: A Call for a Socioecological Approach to AIDS Communication in Sub-Saharan Africa. Health Commun..

[8] Chen L., Yang X. (2019). Using EPPM to Evaluate the Effectiveness of Fear Appeal Messages across Different Media Outlets to Increase the Intention of Breast Self-Examination among Chinese Women. Health Commun..

[9] Cho H. (2006). Fear appeals for individuals in different stages of change: Intended and unintended effects and implications on public health campaigns. Health Commun..

[10] Quick B.L., LaVoie N.R., Reynolds-Tylus T., Martinez-Gonzalez A., Skurka C. (2018). Examining Mechanisms Underlying Fear-Control in the Extended Parallel Process Model. Health Commun..

[11] Sun C., Wang F., Jiang M. (2021). How Can E-Cigarette Fear Appeals Improve the Perceived Threat, Fear, Anger, and Protection Motivation of Young People. Front. Psychol..

[12] Qin C., Wang R., Tao L., Liu M., Liu J. (2022). Acceptance of a third dose of COVID-19 vaccine and associated factors in China based on Health Belief Model: A national cross-sectional study. Vaccines.

[13] Pitts M.J., Stanley S.J., Kim S. (2017). College males’ enduring and novel health beliefs about the HPV vaccine. Health Commun..

[14] Nan X. (2012). Communicating to Young Adults About HPV Vaccination: Consideration of Message Framing, Motivation, and Gender. Health Commun..

[15] Robinson E., Jones A., Lesser I., Daly M. (2021). International estimates of intended uptake and refusal of COVID-19 vaccines: A rapid systematic review and meta-analysis of large nationally representative samples. Vaccine.

[16] WHO COVAX Delivers its 1 Billionth COVID-19 Vaccine Dose. https://www.who.int/news/item/16-01-2022-covax-delivers-its-1-billionth-covid-19-vaccine-dose.

[17] Witte K., Cameron K.A., McKeon J.K., Berkowitz J.M. (1996). Predicting risk behaviors: Development and validation of a diagnostic scale. J. Health Commun..

[18] Cialdini R.B., Reno R.R., Kallgren C.A. (1990). A focus theory of normative conduct—Recycling the concept of norms to reduce littering in public places. J. Personal. Soc. Psychol..

[19] Lapinski M.K., Rimal R.N. (2005). An explication of social norms. Commun. Theory.

[20] Chinese Government Website Press Conference of the Joint Prevention and Control Mechanism of the State Council. http://www.gov.cn/xinwen/gwylflkjz174/index.htm.

[21] Sallam M. (2021). COVID-19 vaccine hesitancy worldwide: A concise systematic review of vaccine acceptance rates. Vaccines.

[22] Cohen J. (2013). Statistical Power Analysis for the Behavioral Sciences.

[23] Chou W.-Y.S., Budenz A. (2020). Considering Emotion in COVID-19 Vaccine Communication: Addressing Vaccine Hesitancy and Fostering Vaccine Confidence. Health Commun..

[24] Nabi R.L., Prestin A. (2016). Unrealistic hope and unnecessary fear: Exploring how sensationalistic news stories influence health behavior motivation. Health Commun..

[25] Vorpahl M.M., Yang J.Z. (2018). Who Is to Blame? Framing HPV to Influence Vaccination Intentions among College Students. Health Commun..

[26] Han B.-C. We Cannot Surrender Reason to the Virus. https://write.as/0hwmokmqr13vm2fw.md?fbclid=IwAR3XwjnxoTBa7QikcF85oW0i0mszNcw7NI_c8yPq3EYp0x9CXaJYq9pbzU&from=timeline.

[27] SteelFisher G.K., Caporello H., McIntosh R., Safdar R.M., Desomer L., Chimenya D., Abdelwahab J., Ratna J., Rutter P., O’Reilly D. (2022). Preventing erosion of oral polio vaccine acceptance: A role for vaccinator visits and social norms. Vaccine.

[28] Agranov M., Elliott M., Ortoleva P. (2021). The importance of Social Norms against Strategic Effects: The case of Covid-19 vaccine uptake. Econ. Lett..

[29] Yajie C., Ye L., Fei S. (2020). Knowledge and Behavior of the Chinese Public Under the COVID-19 Epidemic—An Empirical Study Based on the “National Public Scientific Cognition and Attitude” Survey(Chinese). Shanghai J. Rev..

[30] Shen L. (2017). Putting the fear back again (and within individuals): Revisiting the role of fear in persuasion. Health Commun..

[31] Huang Y., Liu W. (2022). Promoting COVID-19 Vaccination: The Interplay of Message Framing, Psychological Uncertainty, and Public Agency as a Message Source. Sci. Commun..

[32] Malik A.A., McFadden S.M., Elharake J., Omer S.B. (2020). Determinants of COVID-19 vaccine acceptance in the US. eClinicalMedicine.

[33] Litaker J.R., Tamez N., Lopez Bray C., Durkalski W., Taylor R. (2021). Sociodemographic Factors Associated with Vaccine Hesitancy in Central Texas Immediately Prior to COVID-19 Vaccine Availability. Int. J. Environ. Res. Public Health.

[34] Euronews Europe and Central Asia Could See 500 k COVID Deaths by February 1, Says WHO Europe Chief. https://www.euronews.com/2021/11/04/europe-and-central-asia-could-see-500k-covid-deaths-by-february-1-says-who-europe-chief.

[35] National Health Commission of People’s Republic of China Transcript of the Press Conference of the Joint Defense and Control Mechanism of the State Council on 6 November 2021. http://www.nhc.gov.cn/xcs/s3574/202111/28aeb8577b8747ed877f582d50f51035.shtml.

[36] National Health Commission of People’s Republic of China Transcript of the Press Conference of the Joint Defense and Control Mechanism of the State Council on 24 October 2021. http://www.nhc.gov.cn/xcs/s3574/202110/c8145fa890354d2c96fed651c8e41763.shtml.

